# Sequential sampling of visual objects during sustained attention

**DOI:** 10.1371/journal.pbio.2001903

**Published:** 2017-06-28

**Authors:** Jianrong Jia, Ling Liu, Fang Fang, Huan Luo

**Affiliations:** 1 School of Psychological and Cognitive Sciences, Peking University, Beijing, China; 2 IDG/McGovern Institute for Brain Research, Peking University, Beijing, China; 3 Beijing Key Laboratory of Behavior and Mental Health, Peking University, Beijing, China; 4 Peking–Tsinghua Center for Life Sciences, Peking University, Beijing, China; University of Birmingham, United Kingdom of Great Britain and Northern Ireland

## Abstract

In a crowded visual scene, attention must be distributed efficiently and flexibly over time and space to accommodate different contexts. It is well established that selective attention enhances the corresponding neural responses, presumably implying that attention would persistently dwell on the task-relevant item. Meanwhile, recent studies, mostly in divided attentional contexts, suggest that attention does not remain stationary but samples objects alternately over time, suggesting a rhythmic view of attention. However, it remains unknown whether the dynamic mechanism essentially mediates attentional processes at a general level. Importantly, there is also a complete lack of direct neural evidence reflecting whether and how the brain rhythmically samples multiple visual objects during stimulus processing. To address these issues, in this study, we employed electroencephalography (EEG) and a temporal response function (TRF) approach, which can dissociate responses that exclusively represent a single object from the overall neuronal activity, to examine the spatiotemporal characteristics of attention in various attentional contexts. First, attention, which is characterized by inhibitory alpha-band (approximately 10 Hz) activity in TRFs, switches between attended and unattended objects every approximately 200 ms, suggesting a sequential sampling even when attention is required to mostly stay on the attended object. Second, the attentional spatiotemporal pattern is modulated by the task context, such that alpha-mediated switching becomes increasingly prominent as the task requires a more uniform distribution of attention. Finally, the switching pattern correlates with attentional behavioral performance. Our work provides direct neural evidence supporting a generally central role of temporal organization mechanism in attention, such that multiple objects are sequentially sorted according to their priority in attentional contexts. The results suggest that selective attention, in addition to the classically posited attentional “focus,” involves a dynamic mechanism for monitoring all objects outside of the focus. Our findings also suggest that attention implements a space (object)-to-time transformation by acting as a series of concatenating attentional chunks that operate on 1 object at a time.

## Author summary

In a crowded visual scene, attention must be efficiently and flexibly distributed over time and space to accommodate different contexts in a task. Recent studies have proposed that attention is a dynamic process that organizes copious information temporally. However, how the brain coordinates attention among multiple visual objects and whether this dynamic mechanism mediates attention at a general level remain largely unknown. In this study, we analyze electroencephalography (EEG) recordings during a multi-object selective attention task to extract object-specific neuronal responses. We demonstrate that attention rhythmically switches between visual objects every approximately 200 ms. Furthermore, the spatiotemporal sampling profile of attention adaptively changes in various task contexts and correlates with behavioral performance during attention. Our work provides direct neural evidence supporting the idea that multiple objects are sequentially sorted according to their priority in attentional contexts. The results suggest that attention is intrinsically dynamic, acting as a series of concatenating chunks of attention that operate on 1 object at a time to maintain awareness of the whole scene.

## Introduction

The brain has limited processing capacity, yet it is constantly confronted with enormous amounts of information. Attentional mechanisms are therefore needed to selectively enhance the most task-relevant information [1–4]. It is well established that attention increases the neural response to a stimulus so that the attended-to representation is enhanced and wins the competition for the limited neural resources [3,5–9]. These findings presumably imply that, during sustained selective attentional tasks in which subjects are instructed to focus on one location over others, attention would persistently dwell on the task-relevant location.

However, a growing number of studies have taken a different perspective by focusing on the temporal aspects of attention. These experiments propose that, rather than remaining stationary, attention is essentially a highly dynamic and flexible process that organizes and structures complex information in the temporal dimension. For example, even when attention is sustained on 1 location, the stimulus is sampled periodically rather than continuously [10]. Moreover, several recent studies have used a time-resolved psychophysical measurement to disclose rhythms in attentional behavioral performance (i.e., behavioral oscillations), suggesting that multiple locations, features, and objects are sampled alternately in various phases of the attentional rhythm [11–17]. Together, these new findings suggest that sustained attention, in addition to the classically posited attentional “focus,” might contain a dynamic mechanism for monitoring all locations or objects outside the focus [13,18,19]. However, most of the evidence supporting the rhythmic multi-item sampling view is based on behavioral measurements [11,13,14,16,17,20] or pre-target brain activities [19,21], and there is a lack of direct neural evidence for sequential attentional modulation during stimulus processing. Moreover, this sampling mechanism should be flexible enough to accommodate changing behavioral demands, yet it remains unknown how the brain dynamically coordinates attentional resources among multiple objects to efficiently sample the external world in various task contexts. It is also important to know whether the rhythmic sampling mechanism mediates attention at a general level rather than just under certain circumstances (e.g., divided attention).

We employed electroencephalography (EEG), a time-resolved brain technique, to access the spatiotemporal characteristics of attention. Notably, a key challenge of this technique lies in how to disentangle temporally overlapping responses from multiple neuronal sources (e.g., mixed responses from multiple items). In other words, we need to extract the neural activity that exclusively represents a single spatial location or an object in a visual display from the overall EEG responses. The temporal response function (TRF) method, which has recently been used in several auditory studies, is able to dissociate the neural correlates of individual auditory streams from an auditory scene consisting of concurrent speech streams [22–24]. Recently, using similar methods, ongoing visual transients were surprisingly found to elicit long-lasting 10 Hz echoes in neuronal responses [25]. Motivated by these studies, we employed covert sustained visual attentional paradigms in combination with the TRF method to overcome the limitations of EEG and to address the issue.

We are mainly interested in 2 issues: how attention is allocated to multiple visual objects over time during a sustained selective attentional task and whether the spatiotemporal attention profile is flexibly modulated in different task contexts. Our results reveal robust spatiotemporal attention profiles during stimulus processing, characterized by inhibitory alpha-band (approximately 10 Hz) activity switching between attended and unattended locations every 200 ms, suggesting that attention monitors all spatial locations by sampling them in a temporally dissociated way. Moreover, this attentional switching pattern becomes increasingly prominent as the task requires a more uniform distribution of attention over locations, supporting the idea that the spatiotemporal distribution of attention is flexibly adjusted in different contexts. Critically, the neuronal switching pattern correlates with attentional behavioral performance. Finally, this attentional profile is not limited to the spatial dimension but is maintained in a multiple object tracking (MOT) task, suggesting the presence of a general temporal organization mechanism for multi-object attention. Our findings thus speak to a generally central function of sequential sampling in the attentional mechanism.

## Results

**Fig 1:**
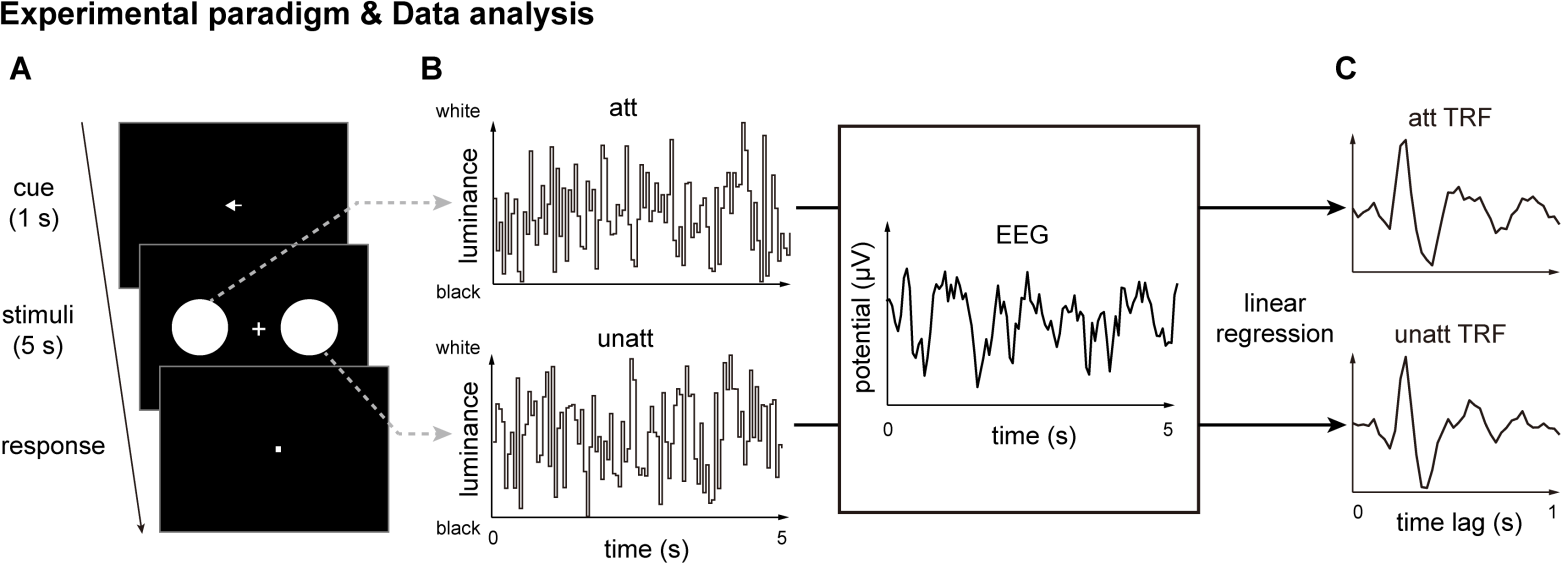
Experimental paradigm for Experiments 1 and 2 and illustration of the temporal response function (TRF) approach. (A) A central arrow cue appeared at the beginning of each trial to indicate which side (left or right) the subject should covertly attend to for subsequent target detection. Two discs were then presented simultaneously in the left and right visual fields for 5 seconds, during which time subjects were instructed to detect the appearance of a target square within the discs by pressing 1 of 2 response keys at the end of each trial. The target occurred at a random time so that subjects had to maintain their attention on the discs. Across trials, the contrast of the target square relative to the momentary disc luminance was adjusted to maintain 80% detection performance. For 100% cue validity (Experiment 1), the target only appeared in the cued disc; for 75% cue validity (Experiment 2), the target appeared in the cued disc 75% of the time and in the uncued disc 25% of the time. (B) The luminance of the 2 discs was independently and randomly modulated throughout the trial, resulting in 2 independent 5 s random temporal sequences (example sequences are shown; top: attended visual stimulus luminance sequence, bottom: unattended visual stimulus luminance sequence). At the same time, electroencephalography (EEG) responses were recorded. (C) The TRF approach was used to calculate the impulse brain response for the attended (top, att) and unattended (bottom, unatt) visual sequences. TRF characterizes the brain response to a unit increase in luminance in a stimulus sequence, with the time axis representing the latency after each transient unit. Note that the att TRF and unatt TRF were derived from the same EEG responses but were separated based on the corresponding stimulus luminance sequence (see panel B).

We recorded 64-channel EEG signals from human subjects fixating on a central spot while 2 discs were displayed simultaneously, 1 in the left and 1 in the right visual field. As shown in Fig 1A, at the beginning of each trial, a central arrow cue (Experiment 1: 100% validity; Experiment 2: 75% validity) indicated which side (left or right) the subjects should attend to for target detection. The target appeared at a random time in 25% of the 5-s trials. Subjects were requested to maintain a central fixation and to detect whether a target square appeared, indicated by pressing 1 of 2 response buttons at the end of each trial. The contrast of the target square (i.e., relative to the luminance of the background disc) was adjusted across trials (using a 3-down-1-up staircase procedure) so that the overall detection performance was maintained at around 80%. In Experiment 1 (100% cue validity), the target only appeared in the cued disc, whereas in Experiment 2 (75% cue validity), the target appeared in the cued disc with 75% probability and in the uncued disc with 25% probability. Subjects were informed of the cue validity before the experiment began.

Critically, the luminance of both the attended and unattended discs was independently modulated for 5 s, resulting in 2 independent 5-s random temporal sequences (Fig 1B). Next, we used the TRF approach [22,24] to calculate and separate neural impulse responses for the attended and unattended visual sequences (att TRF versus unatt TRF) from the same EEG recordings (Fig 1C). The TRF method, which uses linear regression to quantify the stimulus–response relationships, models the neural impulse response function (i.e., the brain response to a unit increase in luminance in a stimulus sequence). This approach has been used in previous studies [22,24,25]. To avoid the influence of the onset EEG response, which may bias the estimated TRF results, we extracted the middle part of the 5-s EEG trial responses (0.5–4.5 s) for further TRF calculation (S1 Fig).

### Experiment 1 (100% cue validity): Attention-elicited alpha inhibition

**Fig 2:**
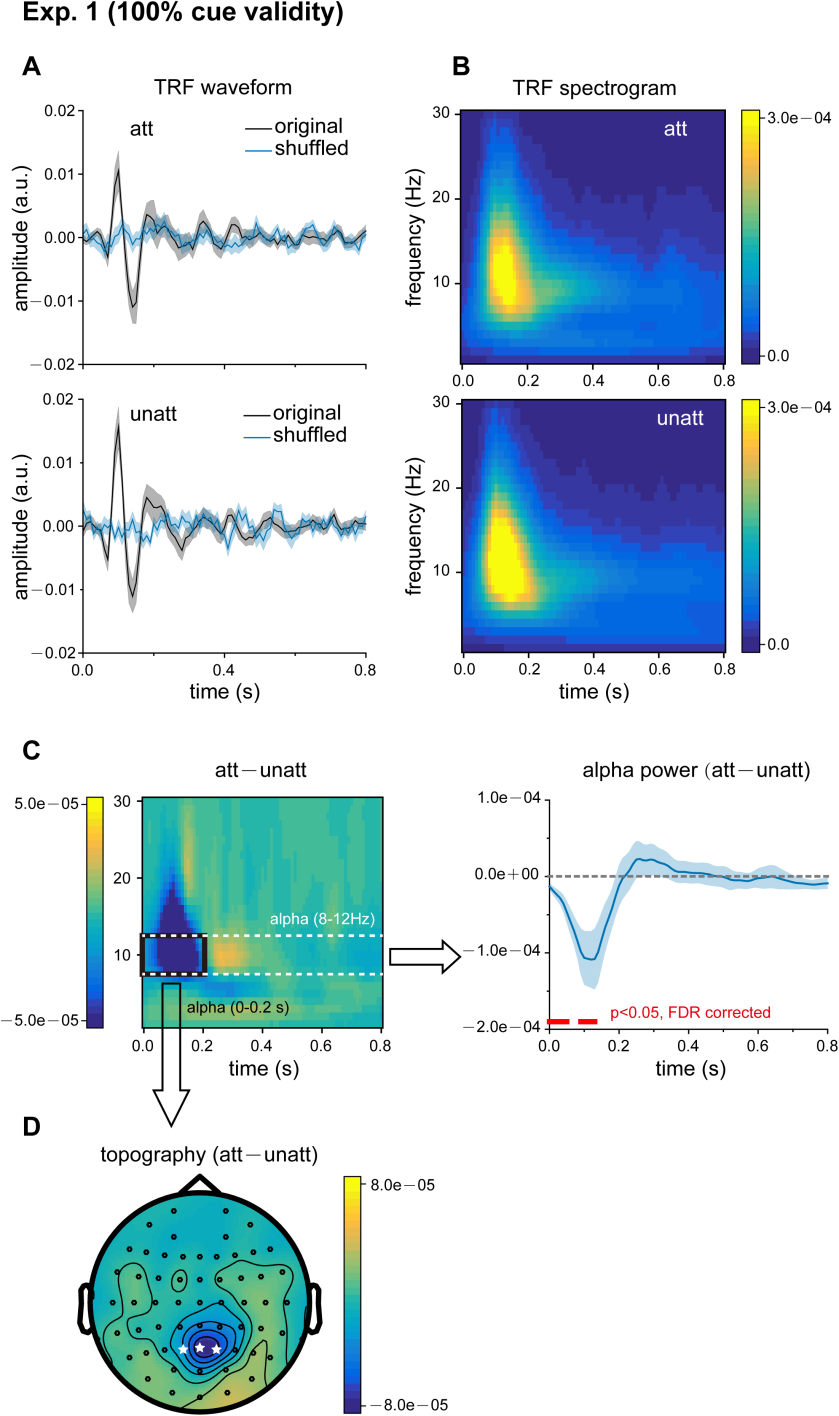
Results for Experiment 1 (100% cue validity): attentional alpha inhibition in temporal response functions (TRFs). (A) Grand average (*N* = 18) TRF waveforms for attended (top panel, black lines, mean ± SEM) and unattended (bottom panel, black lines, mean ± SEM) visual sequences as a function of latency (0–0.8 s), representing brain responses to each unit increase in luminance in the visual stimulus sequence. Blue lines represent the TRFs obtained after the stimulus sequences and electroencephalography (EEG) responses were shuffled. (B) Grand average (*N* = 18) time–frequency power profile for attended (att; top) and unattended (unatt; bottom) TRFs as a function of latency (0–0.8 s) and frequency (0–30 Hz). (C) Left: grand average (*N* = 18) time–frequency plots for att − unatt TRF power difference. Right: grand average (blue lines, mean ± SEM) time course for att − unatt TRF power within the alpha band (8–12 Hz). Red horizontal lines at the bottom indicate points showing significant power differences in the alpha-band between att and unatt (*p* < 0.05, two-tailed, false discovery rate [FDR] corrected). (D) Grand average distribution map for initial 200 ms alpha-band inhibition (mean alpha-band att − unatt TRF power difference within the first 200 ms, black box in Fig 2C). White stars indicate channels with the strongest attentional alpha inhibition effects. The data are provided in the Supporting Information (see S1 Data).

Eighteen subjects participated in Experiment 1 (100% attentional cue validity). Fig 2A illustrates the TRFs for attended (upper panel) and unattended (lower panel) visual sequences (black line), which represent the brain response for each unit increase in luminance in the stimulus sequence as a function of time lag (0–0.8 s). The TRF responses became flat and noisy when the relation between the stimulus sequence and the corresponding trial recordings was shuffled (blue lines), supporting the idea that the estimated TRF represents a genuine stimulus-specific tracking response.

Next, we performed a spectrotemporal analysis on the TRFs to examine their fine dynamic structures as a function of frequency (0–30 Hz) and time (0–0.8 s); this was done for each subject separately. The TRFs for both attended and unattended sequences demonstrated strong alpha-band (8–12 Hz) activation (Fig 2B), consistent with previous results [25]. When comparing the spectrotemporal power profiles between att and unatt TRFs, we observed significant alpha-band inhibition (att < unatt) within the first 200 ms, followed by a rebound trend (att > unatt) in the next 200 ms (Fig 2C). Topographical mapping of the alpha-band inhibition revealed that the attentional effects occurred mainly over parietal electrodes (Fig 2D). In conjunction with the results of previous studies that have revealed the inhibitory function of induced alpha-band activity in spatial attention [26–29], our TRF results (i.e., att–unatt alpha inhibition) suggest that, during selective attention, the unattended visual sequence is inhibited by increasing the alpha activity in the neural impulse response relative to the response of the attended sequence. When we examined the attention-related TRF effects for left and right stimuli separately, we still observed alpha inhibition and a similar topographical distribution (S2 Fig).

### Experiment 2 (75% cue validity): Alpha inhibition followed by alpha rebound

**Fig 3:**
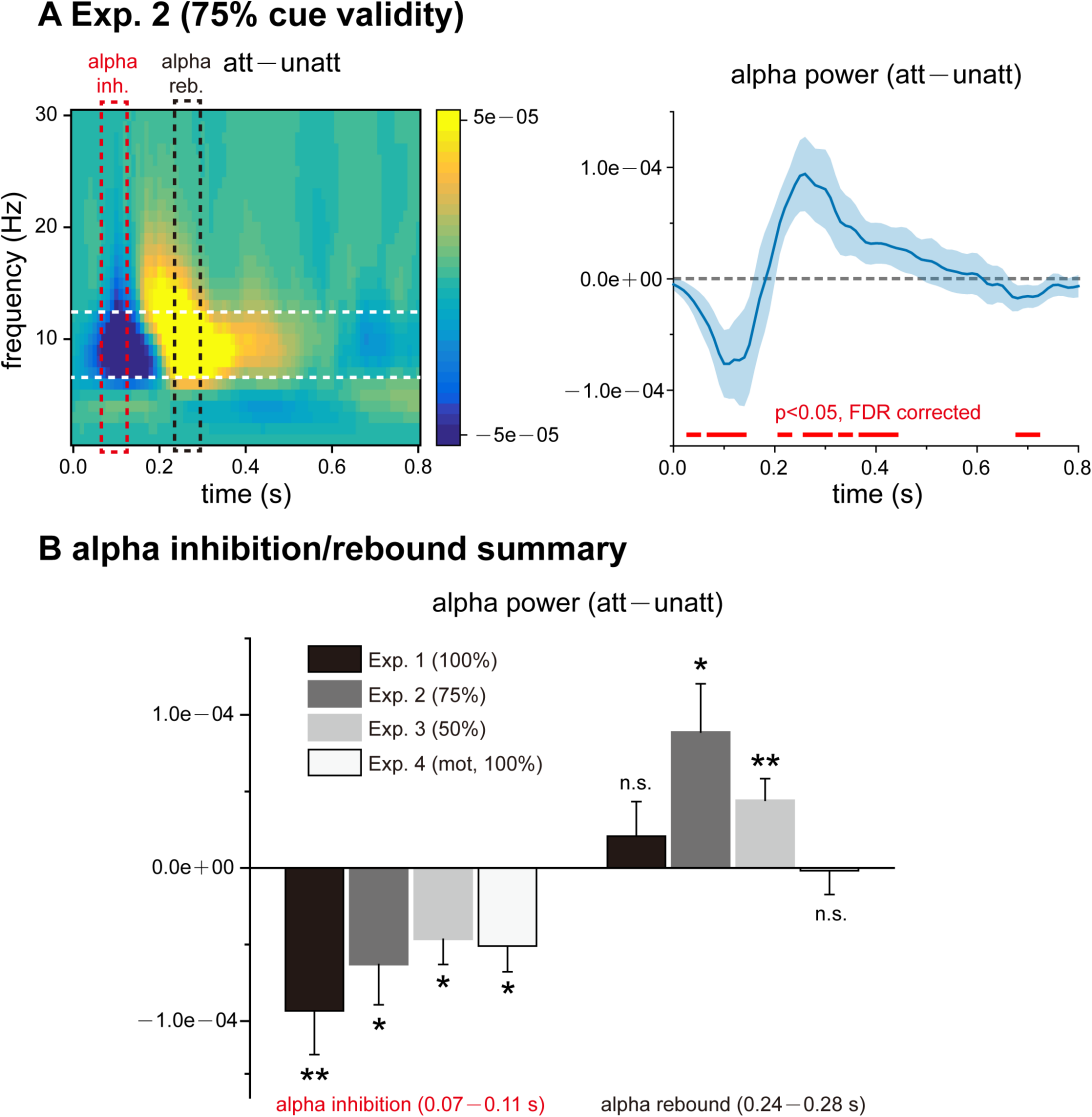
Results for Experiment 2 (75% cue validity) and summary of alpha inhibition and alpha rebound. (A) Temporal response function (TRF) results for Experiment 2 (75% cue validity). Left: grand average (*N* = 20) time–frequency plots for att–unatt TRF power difference. Right: grand average (blue lines, *N* = 20, mean ± SEM) time course for att–unatt TRF power within the alpha band (8–12 Hz). Red horizontal lines at the bottom indicate points showing a significant power difference in the alpha band between att and unatt (*p* < 0.05, two-tailed, false discovery rate [FDR] corrected). Note the emergence of alpha-band rebound right after the alpha-band inhibition, suggesting attentional switching. (B) Grand average att–unatt TRF alpha-band power averaged over an early (alpha inhibition, 0.07–0.11 s, red dotted box in Fig 3A) and a subsequent late (alpha rebound, 0.24–0.28 s, black dotted box in Fig 3A) time range for each experiment. Experiment 1: *N* = 18; Experiment 2: *N* = 20; Experiment 3: *N* = 16; Experiment 4: *N* = 11. * *p* < 0.05, ** *p* < 0.01, *t* test. MOT: multiple object tracking. The data are provided in the Supporting Information (see S2 Data).

The protocol for Experiment 2 was exactly the same as that of Experiment 1 (Fig 1A), except that the attentional cue validity decreased to 75%. Twenty subjects participated in Experiment 2 and were informed of the target distribution probability (i.e., 75%) before the experiment. Similar to Experiment 1, the luminance of the 2 discs was independently and randomly modulated so that the att TRF and unatt TRF could be estimated separately from the same EEG recordings (Fig 1C). Att TRF, compared to unatt TRF, again showed alpha-band inhibition within the first 200 ms (Fig 3A), similar to Experiment 1 (Fig 2C). However, to our surprise, immediately after the initial alpha inhibition (dotted red box), there appeared to be a significant att–unatt alpha rebound during the following 200 ms (dotted black box), indicating that attention switches to the unattended visual object by increasing the inhibitory alpha-band activities on the attended object.

In sum, by examining the TRFs for attended and unattended visual sequences from the same EEG responses in these 2 experiments, we discovered that the neural impulse responses differ between attended and unattended stimuli, as manifested mainly in the alpha-band profiles. Attention first dwells on the attended stimulus by producing inhibitory alpha activity on the unattended stimulus (significant for both Experiment 1 and Experiment 2) and then subsequently switches to the unattended stimulus by exerting inhibition on the attended stimulus (not statistically significant in Experiment 1, statistically significant in Experiment 2). Fig 3B summarizes the initial alpha inhibition and the subsequent alpha rebound for both experiments, as well as for Experiments 3 and 4 (One-sample *t* test; Alpha inhibition: *t* = −3.27, *p* = 0.005, Cohen’s d = −0.77 for Experiment 1; *t* = −2.42, *p* = 0.03, Cohen’s d = −0.54 for Experiment 2; *t* = −2.82, *p* = 0.01, Cohen’s d = −0.71 for Experiment 3; *t* = −3.03, *p* = 0.01, Cohen’s d = −0.91 for Experiment 4; Alpha rebound: *t* = 0.92, *p* = 0.37, Cohen’s d = 0.22 for Experiment 1; *t* = 2.82, *p* = 0.01, Cohen’s d = 0.63 for Experiment 2; *t* = 3.12, *p* = 0.007, Cohen’s d = 0.78 for Experiment 3; *t* = −0.12, *p* = 0.91, Cohen’s d = −0.04 for Experiment 4). Notably, the alpha-rebound effects were different across experiments (two-way ANOVA, Att*experiment interaction effect, F = 2.44, *p* = 0.07, partial η^2^ = 0.11).

A recent study revealed sustained alpha echoes in TRFs and their attentional enhancement effects [25]. We therefore reexamined our results and also observed alpha echoes in the later temporal range of TRFs (0.3–0.6 ms) and an increase in alpha power (att > unatt), but it was not statistically significant (S3 Fig).

What is the reason for the observed difference in the TRFs between the 2 experiments? We hypothesize that the difference in the cue validity (100% versus 75%) in the 2 experiments resulted in different attentional distribution in space, which in turn led to the distinct temporal course of attentional sampling. Specifically, in Experiment 1, with 100% cue validity, subjects assigned all of their attention to the cued object, whereas in Experiment 2, with 75% cue validity, subjects also needed to allocate some attention to the uncued side, because the target had a 25% probability of appearing there. We speculate that it is the spatial extension of attention in Experiment 2 that leads to the enhanced sequential processing of visual stimuli.

### Experiment 3 (50% cue validity): Prolonged alpha alternations over spatial locations

**Fig 4:**
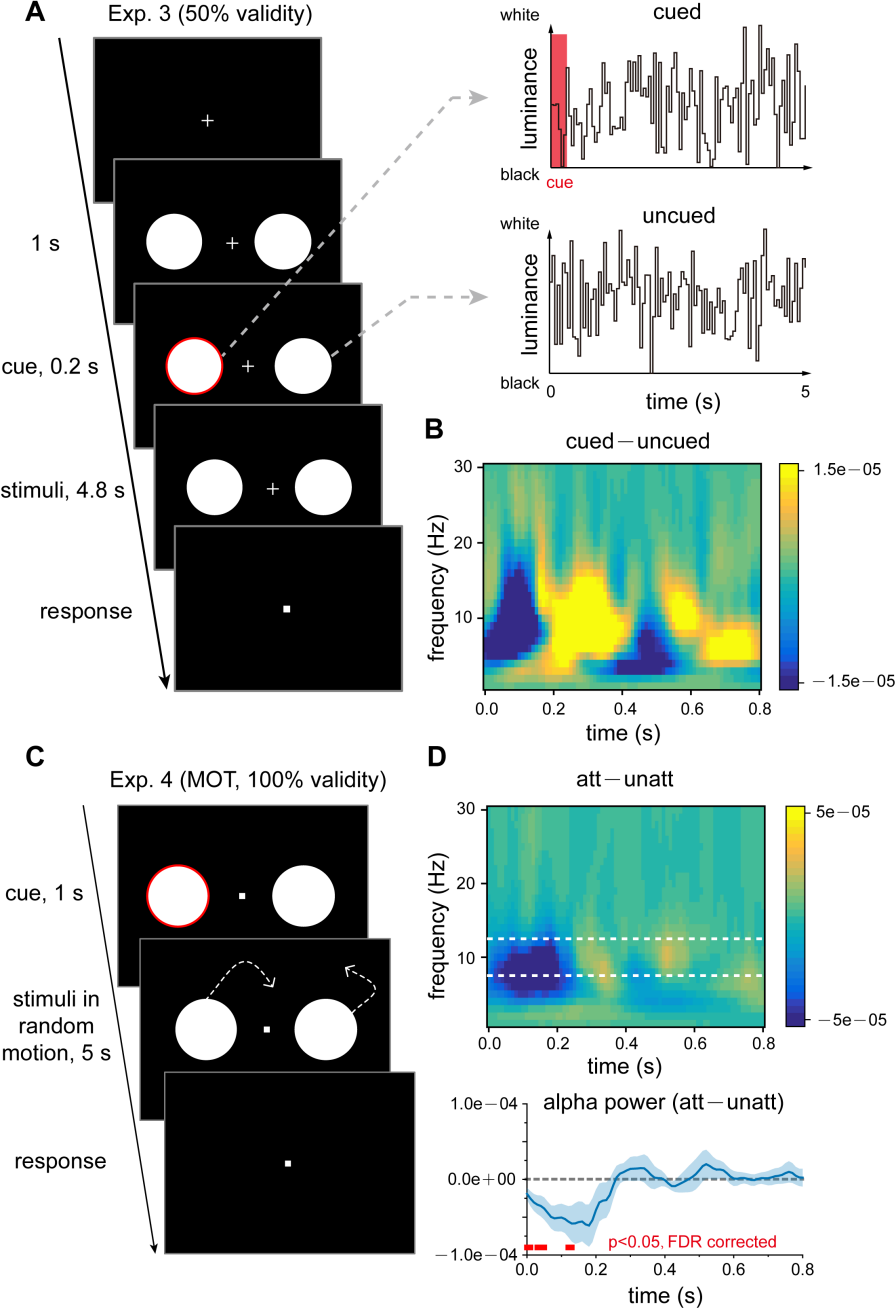
Experiment 3 (50% cue validity) and Experiment 4 (100% cue validity, multiple object tracking [MOT]). (A) In Experiment 3 (50% cue validity), subjects fixated on a central point and covertly attended to 2 discs presented in the left and right visual fields for target detection. Subjects were instructed to simultaneously pay attention to both discs and were informed that the target would be equally likely to appear within the discs and that the initial cue would not predict the target location. After a noninformative red circle cue (cue validity: 50%) appeared around 1 of the 2 discs, the luminance of the 2 discs was independently and randomly modulated for 5 s (top: cued visual sequence; bottom: uncued visual sequence), during which time subjects were instructed to monitor a randomly occurring target. (B) Grand average (*N* = 16) time–frequency plots for cued–uncued TRF power difference in Experiment 3 (cue validity: 50%). Note the prolonged alpha-band switching (blue–red pattern), suggesting that attentional shifting is enhanced when attention is evenly distributed across the 2 spatial locations (50% cue validity). (C) In Experiment 4 (MOT experiment), a red circle cue at the beginning of each trial indicated which disc the subjects should covertly attend to for subsequent target detection. The 2 disks were then moved randomly and smoothly across the screen for 5 s, during which time the subjects were instructed to detect the appearance of a target within the cued disc. Here, the cue validity was 100%, which means that the target only appeared in the cued disk, similar to Experiment 1. (D) Experiment 4 results. Top: Grand average (*N* = 11) time–frequency plots for att–unatt TRF power difference. Bottom: grand average (blue lines, *N* = 11, mean ± SEM) time course for att–unatt TRF power within the alpha band (8–12 Hz). Red horizontal lines at the bottom indicate points showing significant power differences in the alpha band between att and unatt (*p* < 0.05, one-tailed, false discovery rate [FDR] corrected). Note the initial alpha inhibition followed by an alpha rebound trend, similar to Experiment 1 (see Fig 2C). The data are provided in the Supporting Information (see S3 Data).

Next, we investigated whether this attentional switching pattern becomes stronger when subjects allocate attention to the 2 locations more evenly. As shown in Fig 4A, we used an exogenous pre-cuing attentional paradigm [30,31], in which subjects (*N* = 16) were asked to covertly attend to the 2 discs simultaneously for target detection. Specifically, a cue that was noninformative as to the subsequent target location (red ring) appeared in 1 of the 2 discs at the beginning of each trial, thereby determining which location would obtain the initial attentional benefit [31]. However, the target was equally likely to occur in both locations (50% cue validity). We then examined the temporal relationship in the TRFs between the cued and uncued stimulus sequences.

The cued TRF, compared to the uncued TRF, showed an alpha-band pattern of inhibition followed by rebound within the first 400 ms (Fig 4B), replicating the results of Experiment 1 and Experiment 2 (Fig 2C and Fig 3A). Notably, another sequence of alpha inhibition followed by a rebound then appeared after the first 400 ms, indicating that the cued–uncued alpha-band alternation became more enduring over time. Thus, attentional switching between locations becomes more temporally persistent when attention is instructed to evenly distributed across the 2 spatial locations (50% cue validity).

Moreover, the 2 disc stimuli in all 3 experiments were presented in the left and right visual fields, and therefore, the observed attentional switching could have been solely caused by interhemispheric competition [32]. To address this issue, we ran a control experiment (*N* = 13) in which the 2 discs were presented in the upper and lower visual fields within the same visual hemifield (S4 Fig). The same alpha-band alternating pattern was observed, thus arguing against the interpretation of interhemispheric competition.

### Experiment 4 (100% cue validity, MOT): Object-based alpha sampling

After establishing the sensitivity of the alpha-band activity in the TRFs to spatial attention (i.e., attention to multiple spatial locations) in the 3 experiments, we asked whether the observed attention-related inhibitory alpha effects could be extended to object space. We employed an MOT paradigm to address this issue [33,34]. As shown in Fig 4C, at the beginning of each trial, a cue (red ring, 100% cue validity) indicated which disc the subjects (*N* = 11) should attend to for target detection. The 2 discs, whose luminance was again randomly modulated in time, then underwent random smooth motion across the screen for 5 s, from which the TRF was calculated for the attended and unattended discs. Interestingly, we observed similar att–unatt alpha inhibition in the TRFs during the first 200 ms, as well as a subsequent nonsignificant rebound in alpha power (Fig 4D), parallel to the findings in Experiment 1 (Fig 2C, 100% cue validity). The results thus suggest that the observed alpha-based profile is not confined to space-based attention but presumably reflects a more general mechanism underlying object-based attention.

### Relation to attentional behavioral effects

**Fig 5:**
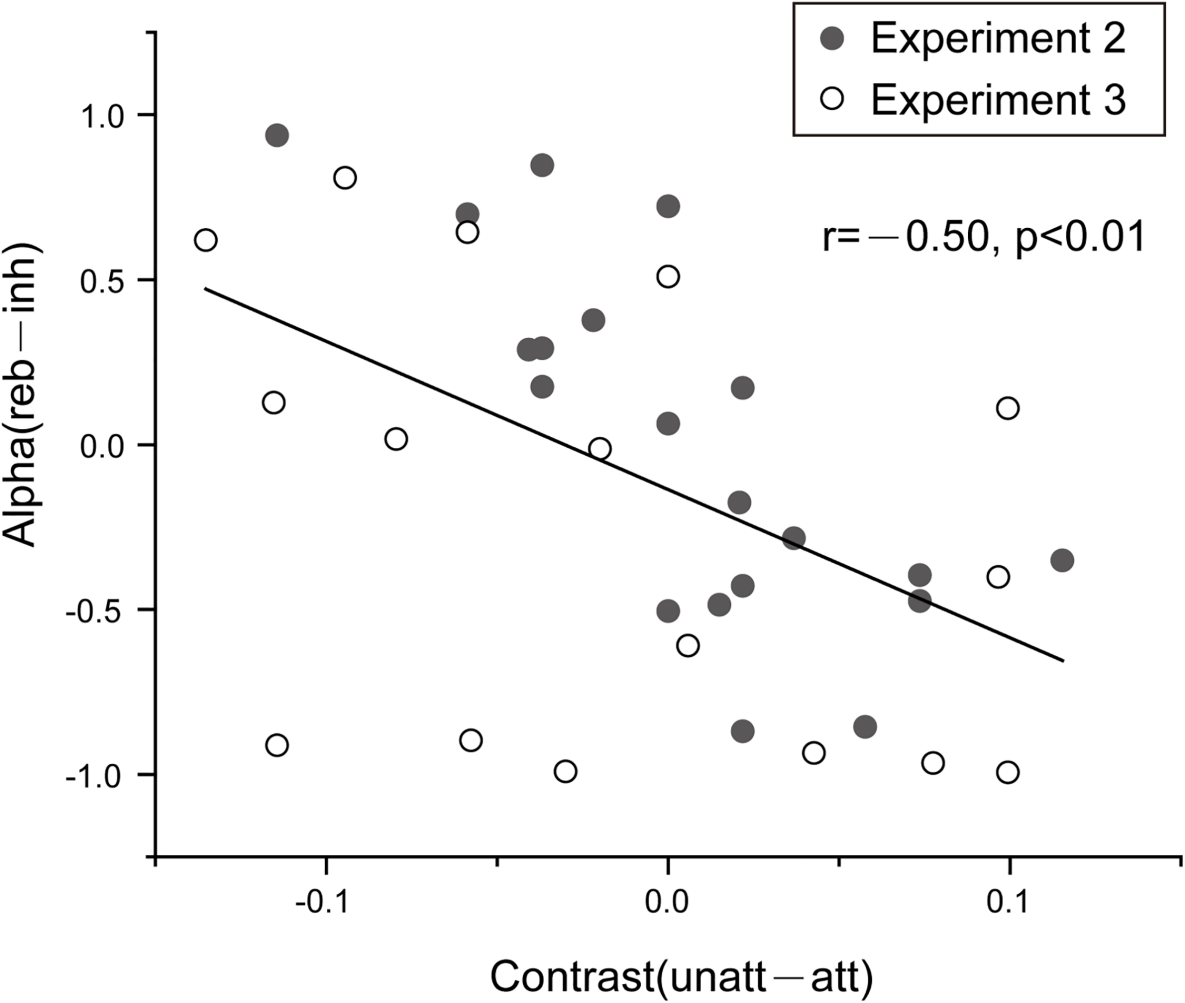
Alpha inhibition-rebound effects relate to attentional behavior. Correlations between behavioral (behavioral index, BI) and alpha switching (neuronal index, NI) measures across participants (Experiment 2: *n* = 20, disc; Experiment 3: *n* = 16, circle). BI: Contrast_unatt_−Contrast_att_; NI: alpha_reb_−alpha_inh_. The negative correlations indicate that, as the unattended object obtained more attentional behavioral benefit (smaller BI), the alpha rebound effects became stronger (larger NI). The data are provided in the Supporting Information (see S4 Data).

Finally, to further evaluate the role of the alpha-band switching profile (i.e., alpha inhibition followed by rebound) in attentional allocation, we calculated the correlation coefficients between the behavioral (behavioral index, BI) and TRF (neuronal index, NI) results across individual subjects. Specifically, the BI for each subject was calculated by comparing the adjusted target contrast between attended and unattended (Contrast_unatt_−Contrast_att_), and the NI was represented by calculating the difference between the alpha inhibition and subsequent alpha rebound in the TRFs (alpha_reb_−alpha_inh_). As shown in Fig 5, they showed a strong negative correlation (combining Experiment 2 and Experiment 3, r = −0.5, *p* < 0.01). Specifically, as the unattended object obtained more attentional benefit reflected in behavior (lower target contrast, smaller BI), the alpha rebound effect, which represents the alpha-mediated attentional switching to the unattended object, became increasingly stronger (larger NI).

## Discussion

In this study, we used covert sustained attentional paradigms in combination with a TRF approach to examine how attention is distributed over multiple locations (or objects) in systematically manipulated task contexts. First, we found that attentional effects are robustly encoded by an initial alpha-band decrease in the neural impulse response, indicating that, during ongoing processing, attention first stays on the attended item by enhancing the inhibitory alpha power on the unattended item (Experiments 1–4). Second, around 200 ms after the initial alpha inhibition, attention reverses and somewhat shifts to the unattended item by inhibiting the attended item, even when subjects are instructed to largely or completely focus on the attended item (Experiments 1, 2, and 4), thus indicating a sequential sampling process. Third, the attentional switching pattern becomes increasingly prominent and enduring as the task requires attention to be more uniformly distributed over locations (Experiments 2 and 3). Finally, these switching neuronal patterns correlate with attentional behavioral performance across subjects (Fig 5). In sum, the results demonstrate that attention essentially processes multiple objects alternately in time instead of dwelling on the required-to-be-attended object continuously, and this attentional spatiotemporal profile adaptively changes to accommodate different task contexts. Furthermore, our results suggest that a sequential sampling mechanism is engaged in the general attentional process, through which multiple visual objects are temporally sorted according to their priority in different attention contexts.

We used a TRF method to extract item-based neural responses. Specifically, each item of interest (e.g., a location or an object) was independently modulated by a temporal sequence randomly generated in each trial, and linear regressions were used to model the stimulus–response mapping. TRF thus enables the neural representation that exclusively and robustly follows the specific item to be extracted from the noisy EEG signal. Notably, instead of representing the brain response over the course of a trial, the TRF actually represents the neural impulse response, with the time axis representing the latency after each transient unit in the stimulus. Specifically, each stimulus frame of the temporal sequence acts as an onset probe to assess the associated elicited response. The computed TRF response, by counting all transients throughout the whole trial, would then characterize the brain response for each transient unit during ongoing stimulus processing at the attended location (att TRF) or the unattended location (unatt TRF). Moreover, after removing the grand-averaged evoked TRF responses, we observed a similar alpha-mediated switching pattern (S5 Fig), thus indicating that the observed spectrotemporal patterns are not merely caused by the time-locked components.

In Experiments 1 and 2, when subjects were instructed to attend largely to 1 location, each transient unit at the attended location was processed earlier (alpha inhibition within the first 200 ms) than at the unattended location (alpha rebound within the next 200 ms). In Experiment 3, when the 2 locations were equally task-relevant, the TRF responses mainly reflect the initial attentional capture effects at the cued location. Specifically, given the independent random modulation of luminance at the 2 locations over the course of each trial, the overall influence of the ongoing luminance transients on TRF responses would then be balanced between the 2 locations, leaving the TRF responses dominated by the initial attentional capture effects. Consequently, the cued and uncued locations in Experiment 3 would correspond to the attended and unattended locations in Experiments 1 and 2, respectively, but with different attentional distributions (50% versus 100% and 75%). Taken together, all 3 experiments (1–3) revealed an att-followed-by-unatt sequential activation profile, suggesting that attention sorts neural representations of multiple locations along the temporal dimension according to their attentional priority in various task contexts.

Most importantly, our results support a generally central function of the sequential sampling mechanism in attention. First, the sequential sampling mechanism not only mediates attentional processing when 2 locations are equally weighted in attention (50% cue validity, Experiment 3), consistent with previous work [11,13,14,16,17], but is also essentially engaged in classical selective attentional paradigms, during which time attention focuses mainly on one location over the other (100% cue validity, 75% cue validity, Experiments 1 and 2). Second, the sequential mechanism not only organizes attentional allocation over multiple spatial locations but also mediates the sampling process in object-based space (Experiment 4). Finally, by systematically manipulating attentional contexts (different cue validities, Experiments 1–3), we observed a covarying modulation in the sequential sampling profile. Thus, the results not only suggest that the brain adaptably adjusts the temporal dynamics of attentional allocation among objects to optimize performance but also further confirms the essential connections between sequential sampling and attention. In sum, during sustained attention tasks, when subjects mainly focus on 1 of many items throughout the trial, attention still intrinsically and rhythmically allocates resources to the out-of-focus items. This mechanism makes sense, given that, to survive in a complex environment, it is important to monitor all potentially relevant parts of the world, instead of solely focusing on the task at hand. This view is also in line with a recent model proposing an oscillation-based temporal organization mechanism for processing task-irrelevant inputs [18,35].

Furthermore, our results constitute direct and novel neural evidence for the rhythmic attention view [10,21]. Several previous studies based on behavioral measurements [11,13,16], including our own [14,17], have demonstrated rich oscillatory profiles in attentional performance (i.e., behavioral oscillations), implying that attention rhythmically monitors multiple locations, features, or objects. Recently, an important magnetoencephalography (MEG) study showed that the ongoing pre-target gamma-band activities were out of phase in the theta band (4 Hz) for detected versus missed targets [19], which also supports the rhythmic attention view. However, the results mainly focus on pre-target baseline activities that essentially represent ongoing background signals; hence, there is still a lack of direct neural evidence for whether and how the brain temporally mediates multiple visual objects during ongoing stimulus processing. Instead, our results demonstrate that the brain organizes multiple visual objects in time (i.e., sequential sampling) according to their attentional priority (i.e., attended before unattended) during ongoing stimulus processing, thus providing direct neural evidence. Furthermore, our results show that the sequential sampling profile adaptively changes to accommodate different attentional contexts, further confirming their close connections.

Another key aspect of our observations concerns the inhibitory role of alpha-band activity in attention. It is acknowledged, with evidence from both human and animal studies, that alpha-band responses are closely related to inhibitory functions in various cognitive processes [26–29]. A recent study even disclosed inhibitory alpha-band pulses in behavioral performance during attentional tasks [17]. The inhibitory nature of alpha-band activity has been proposed as a mechanism that converts spatial representations into a temporal code by periodically gating the information flow [35–37]. Meanwhile, a recent experiment revealed long-lasting alpha activity in the TRF responses (“alpha echoes”), which are indeed enhanced by attention [25]. We speculate that the difference between their results and ours (inhibition versus enhancement) mainly occurred because we examined the dynamic structure of the alpha activity, whereas the previous study focused on the overall alpha-echo power averaged over time; indeed, it is quite plausible that both effects coexist (e.g., see S3 Fig). Furthermore, previous pioneering EEG work has revealed that the phase of pre-target alpha-band activity modulates subsequent visual perception, which mainly reflects the extent of temporal alignment between the target and the background attentional excitatory state. Here, we examined the post-stimulus responses instead, and our findings indicate that inhibitory alpha-band activity mediates the multi-object sequential sampling process, such that each visual object is sequentially selected by enhancing the inhibitory alpha-band power on the others.

Low-frequency rhythms, such as the delta and theta bands, have been postulated to constitute the cycle of attentional selection by periodically modulating cortical excitability and segmenting inputs into temporal chunks [38–42]. In support, several recent psychophysical studies have demonstrated rhythmic attentional sampling in behavioral performance [11–14,16,17] and long serial-dependence effects in visual perception [43]. In this study, the attentional selection cycle, consisting of initial alpha inhibition and subsequent alpha rebound, lasted approximately 400 ms, which also corresponds to a low-frequency rhythm. Furthermore, we found that low-frequency attentional switching was maintained in object-based attention, also consistent with a recent behavioral oscillation study [11,13].

In conclusion, the neural mechanisms of sustained attention are usually investigated by assessing how attention modulates overall neuronal responses or pre-stimulus neuronal activity. Here, we provide novel and direct neural evidence that, instead of dwelling on 1 item in a stationary way, attention explores multiple visual objects in a “serial-like” manner by producing inhibitory alpha-band activity to each object in turn. Moreover, the spatiotemporal sampling profiles are modulated adaptively in various task contexts and correlate with behavioral performance. Our results imply the discrete nature of attention [10,44,45] and suggest that attention acts as a temporal sequence concatenated by discrete, alpha-mediated attentional chunks, each of which operates on 1 object at a time, conceptually similar to the discrete chunking model proposed for working memory [46–48]. Finally, and importantly, our study supports a generally central function of temporal organization mechanism in multi-object attention.

## Materials and methods

### Participants

Seventy-eight adults aged 18–24 were recruited from Peking University. Eighteen subjects participated in Experiment 1; 20 participated in Experiment 2; 16 participated in Experiment 3; 13 participated in the control experiment for Experiment 3; and 11 participated in Experiment 4. Some subjects attended more than 1 experiment. All participants had normal or corrected-to-normal vision and had no history of psychiatric or neurological disorders. All experiments were carried out in accordance with the Declaration of Helsinki. All participants provided written informed consent prior to the start of the experiment, which was approved by the Research Ethics Committee at Peking University (2015-03-05c2).

### Stimuli and tasks

Subjects sat in a dark room in front of a CRT monitor (100 Hz refresh rate), and their heads were stabilized using a chin rest. In each trial, subjects were requested to maintain fixation on a central point and to covertly monitor the appearance of a target square (side length of 3.75° in Experiment 1–3; side length of 3° in Experiment 4) within 1 of the 2 peripheral discs (5.5°) that were presented at 7.5° to either side of the fixation point (Fig 1A). The target square was presented for 0.5 s and occurred at a random time between 0.25 s and 4.25 s of the 5 s trial in 25% of the trials. At the end of each trial, subjects pressed 1 of 2 buttons to report whether they had detected the target. Across trials, the contrast of the target square (i.e., target luminance relative to the momentary background disc luminance) was adjusted according to the detection accuracy (using a 3-down-1-up staircase procedure), so that the overall target detection performance was maintained at around 80%.

All subjects were instructed to keep the number of eye blinks to a minimum during the experimental trials. Eye movements were monitored using an EyeLink 1000 eye tracker (SR Research), and fixation was required within a 1° visual angle of the fixation point to initiate the experimental trials. The results showed that the participants maintained good fixation on the central cross (within 1°) throughout the experimental trials.

#### Experiments 1 and 2

At the beginning of each trial, a central arrow cue (1-s duration) was presented to indicate the disc (left or right) within which the target would appear later. In Experiment 1 (cue validity of 100%), the target would only be presented in the cued disc and never in the uncued disc. Subjects were informed of the cue validity and thus were aware that they only needed to covertly attend to the cued disc for target detection while ignoring the other disc throughout the 5-s trial. Experiment 2 was the same as Experiment 1, except that the cue validity was 75%. The target appeared in the cued disc with a 75% probability and in the uncued disc with a 25% probability. Subjects were also informed of the cue validity before the experiment.

#### Experiment 3

In Experiment 3, the target occurred in the 2 discs with equal probability, and therefore, subjects had to attend to both discs simultaneously for target detection. At the beginning of each trial, a red ring, serving as a noninformative peripheral cue, appeared for 0.1 s around 1 of the 2 discs so that attention was reset to the corresponding disc [31]. Subjects were informed that the cue in the experiment was 50% in validity and would not predict the target location. In the control experiment for Experiment 3, the 2 discs (side length of 3.75°) were presented on 1 side of the fixation point (either left or right) and above and below the horizontal meridian (4.5° in radius at 10.6° in eccentricity) (S4 Fig, left side).

#### Experiment 4

Experiment 4 was the same as Experiment 1, except that, after the central cue, the 2 discs moved constantly (19.2°/s) across the screen in random directions (Fig 4C). The disc bounced to reverse direction when it touched the edge of the screen or the other disc. The cue validity was 100%, which means that the target would only be presented in the cued disc and would never occur in the uncued disc. Subjects were informed of the cue validity and only needed to covertly attend to the cued disc for target detection while ignoring the other disc throughout the 5-s trial.

#### Luminance modulation

To extract the object-based impulse response using the TRF technique, the luminance of the 2 discs was independently modulated in time each time the frame refreshed (100 Hz monitor refresh rate), according to 2 randomly generated temporal sequences. The CRT refresh rate of 100 Hz allowed us to present temporal frequencies ranging between 0 and 50 Hz. Each randomly generated sequence was tailored to have equal power at all frequencies by normalizing the amplitudes of its Fourier components before applying an inverse Fourier transform. Luminance sequences ranged from black (0 cd/m^2^) to white (84.6 cd/m^2^).

### EEG recording

EEG was recorded continuously using 2 BrainAmp amplifiers and a 64-channel ActiCap (BrainProducts). Horizontal and vertical electrooculograms were recorded by 2 additional electrodes around the subjects’ eyes. EEG data were offline band-pass filtered between 2 and 50 Hz. Independent component analysis was performed independently for each subject to remove eye-movement and artifact components, and the remaining components were back-projected onto the EEG electrode space. All channels were then referenced to the average value of all channels. The EEG was downsampled to 100 Hz before TRF estimation with the stimulus sequences. The stimulus time points for the 4 s before the last 0.5 s of the sequence were entered in the TRF estimation, which was computed at all lags between 0 and 0.8 s. The selection of EEG time points was intended to eliminate the influence of onset and offset responses on the TRF computation.

### Data analysis

#### TRF computation

The mapping between the stimuli luminance input and the recorded EEG data output were analyzed using the multivariate temporal response function (mTRF) toolbox [23,24]. The TRF describes the brain’s linear transformation of a stimulus input, S(t), to the neural response output, R(t), as R(t) = TRF * S(t), where * denotes the convolution operator. Specifically, the TRF computations were performed by a regularized linear regression between the stimulus luminance value and EEG amplitude. A parameter (lambda) was used to control overfitting in the ridge regression. The lambda value was set to 1 for all subjects in the present experiments. Note that the TRF represents an average measure of how the brain responds to a unit change in luminance as a function of latency. The stimulus luminance values and EEG signals were concatenated across trials and then normalized before TRF calculation, so the TRFs were in arbitrary units.

#### Time–frequency analysis

The obtained TRF responses were then analyzed with MATLAB (MathWorks, Inc., Natick, Massachusetts), using the FieldTrip toolbox [49] and wavelet toolbox functions to examine their spectrotemporal power profiles. We analyzed the TRFs for each condition and for each channel within each subject.

To assess the TRF profiles as a function of time (latency of 0–0.8 s) and frequency (0–30 Hz), the TRF temporal profile for each condition was transformed using the continuous complex Gaussian wavelet transform (Wavelet toolbox, MATLAB), with frequencies ranging from 1 to 30 Hz in increments of 1 Hz. The power profiles of the TRFs (squared absolute value) as a function of time and frequency were then extracted from the output of the wavelet transform. Differences in the power profile between the att and unatt conditions were calculated. This time–frequency analysis was performed for each condition, for each channel, and for each subject separately. The grand mean of time–frequency power was then averaged across subjects.

Based on the scalp distribution of the alpha power difference between the att and unatt conditions in the first 200 ms (e.g., Fig 2D shows the results of Experiment 1), data from electrodes Pz, P1, and P2 were selected for further analysis in Experiments 1, 2, and 4. In Experiment 3 and its control experiment, the CPz, Cz, and FCz electrodes showed more prominent effects and were selected for further analysis.

#### Correlation between behavior and alpha switching effects

Two indices (BI and NI) were calculated for each subject. Data from Experiment 2 and Experiment 3 were combined to increase the sample size. Experiment 1 was not included because the target only occurred in the attended side (100% cue validity) and the behavioral results for the unattended condition could not be examined.

Specifically, the BI, which characterizes the attentional behavioral difference between attended and unattended conditions, was calculated as BI = Contrast_unatt_−Contrast_att_, within which the contrast refers to the adjusted contrast for the target when it occurred in the attended or unattended object. The NI, which characterizes the normalized difference between alpha rebound and alpha inhibition in the TRF results, was calculated as $NI=\frac{\left|reb|-|inh\right|}{\left|reb|+|inh\right|}$. A correlation analysis between BI and NI across subjects was then performed.

#### Statistical procedures

To validate that the calculated TRFs were not artifacts, a randomization procedure was used to shuffle the relationship between trial EEG responses and the corresponding stimulus temporal sequence (Fig 2A). In the point-by-point statistics of the time course shown in Fig 2C, Fig 3A and Fig 4D, we corrected all repeated tests in an analysis using the false discovery rate [50]. The alpha inhibition and subsequent alpha rebound shown in Fig 3B were extracted from the average around the trough (70~110 ms) and the peak (250~290 ms) of the alpha power time course. Statistical differences in alpha inhibition and rebound were examined with *t* tests against 0. Paired *t* tests were used to compare the magnitude of the alpha echo between the conditions shown in S3 Fig. All error bars and intervals reflect the standard error of the mean across subjects.

## Supporting information

S1 Fig Trial-averaged ERP responses. Top: ERP waveforms as a function of time (0–5 s). Bottom: Distribution map for the initial ERP onset response, mainly derived from posterior visual channels. Note that to avoid the influence of the onset and offset response, which may bias the estimated temporal response function (TRF) results, we extracted the middle part (red rectangle) of the 5-s EEG trial responses (0.5–4.5 s) for further TRF calculation. The data are provided in the Supporting Information (see S5 Data).
(TIF)

S2 Fig Control analysis for induced alpha & attentional effects in TRFs for left and right stimuli separately. (A) Left: Power spectrum of the EEG during the 5-s stimulus presentation. Right: Spatial distribution of induced alpha-band power. (B) Spatial distribution of induced alpha-band power difference between trials where subjects attended to the left disc and trials where subject attended to the right disc. Note the alpha-band inhibition in contralateral channels, consistent with previous findings. (C) Grand average time-frequency plots for attended (att)–unattended (unatt) TRF power difference (top) and the distribution map for the initial 200 ms alpha-band (dotted white box) inhibition (bottom), for discs presented in the left visual field. (D) Same as C but for discs presented in the right visual field. Note the similar initial alpha-band inhibition and similar spatial distribution to those in Fig 2C & 2D. The data are provided in the Supporting Information (see S6 Data).
(TIF)

S3 Fig Control analysis for alpha echoes. (A) Alpha echoes in Experiment 1. Grand average time-frequency power profile for attended (att) TRF (left) and unattended (unatt) TRF (right), as a function of latency (0.3–0.8 s) and frequency (0–30 Hz). (B) Alpha-echo power (averaged from 0.3–0.6 s, dotted black rectangle) for att (black bar) and unatt (white bar) TRFs. (C) Same as (A) but for Experiment 2. (D) Same as (B) but for Experiment 2. Note the larger overall alpha echoes in both experiments, similar to the results of VanRullen and MacDonald (2012). The data are provided in the Supporting Information (see S7 Data).
(TIF)

S4 Fig Control experiment for Experiment 3. Left: Subjects fixated on a central point and covertly attended to two discs presented in the upper and lower visual field, within the same visual hemifield. Subjects were instructed to pay attention to the two discs simultaneously and were informed that the target was equally likely to appear within both and that the initial cue would not predict the target location. After an uninformative red circle cue (cue validity: 50%) appeared in one of the two discs, the luminance of the two discs was independently and randomly modulated for 5 seconds. Right: Grand average (N = 13) time-frequency plots for cued–uncued TRF power difference. Note the same alpha-band alternating pattern, thus arguing against the interpretation of interhemispheric competition for Experiment 3. The data are provided in the Supporting Information (see S8 Data).
(TIF)

S5 Fig Time-frequency power profile (Att-Unatt) after removing evoked components. Left: Experiment 1 (cue validity: 100%; N = 18); Middle: Experiment 2 (cue validity: 75%; N = 20); Right: Experiment 1 (cue validity: 50%; N = 16). The data are provided in the Supporting Information (see S9 Data).
(TIF)

S1 Data Data for Fig 2. (XLSX)

S2 Data Data for Fig 3. (XLSX)

S3 Data Data for Fig 4. (XLSX)

S4 Data Data for Fig 5. (XLSX)

S5 Data Data for S1 Fig. (XLSX)

S6 Data Data for S2 Fig. (XLSX)

S7 Data Data for S3 Fig. (XLSX)

S8 Data Data for S4 Fig. (XLSX)

S9 Data Data for S5 Fig. (XLSX)

## Abbreviations

BI: behavioral index
EEG: electroencephalography
FDR: false discovery rate
MEG: magnetoencephalography
MOT: multiple object tracking
NI: neuronal index
TRF: temporal response function
