# Supplementary figures and images for: Sequential sampling of visual objects during sustained attention
Source: PLoS Biol. 2017 Jun 28;15(6):e2001903. doi: 10.1371/journal.pbio.2001903 (PMC5489144; doi:10.1371/journal.pbio.2001903)

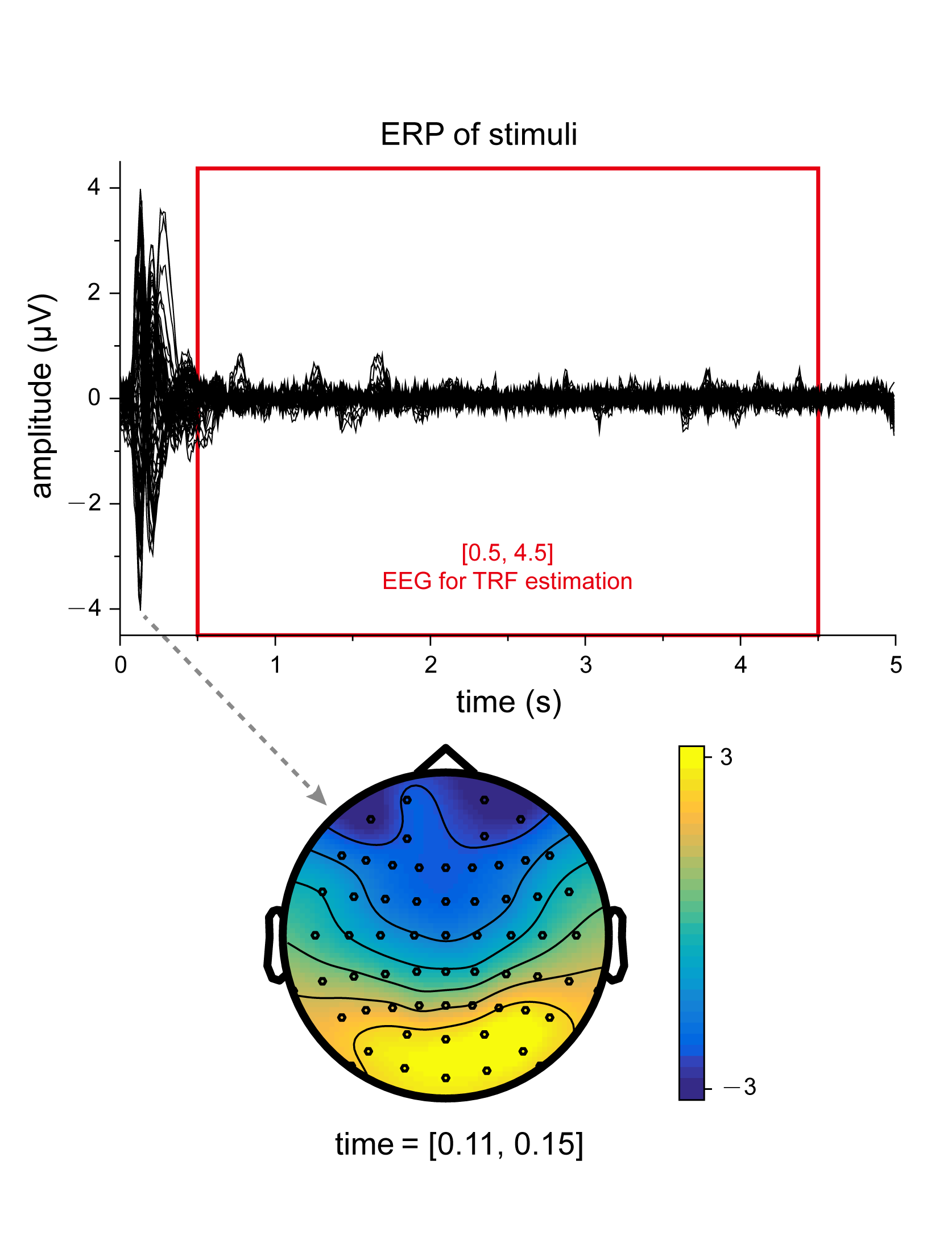

Supplement: S1 Fig — Top: ERP waveforms as a function of time (0–5 s). Bottom: Distribution map for the initial ERP onset response, mainly derived from posterior visual channels. Note that to avoid the influence of the onset and offset response, which may bias the estimated temporal response function (TRF) results, we extracted the middle part (red rectangle) of the 5-s EEG trial responses (0.5–4.5 s) for further TRF calculation. The data are provided in the Supporting Information (see S5 Data). (TIF) [file pbio.2001903.s001.tif]

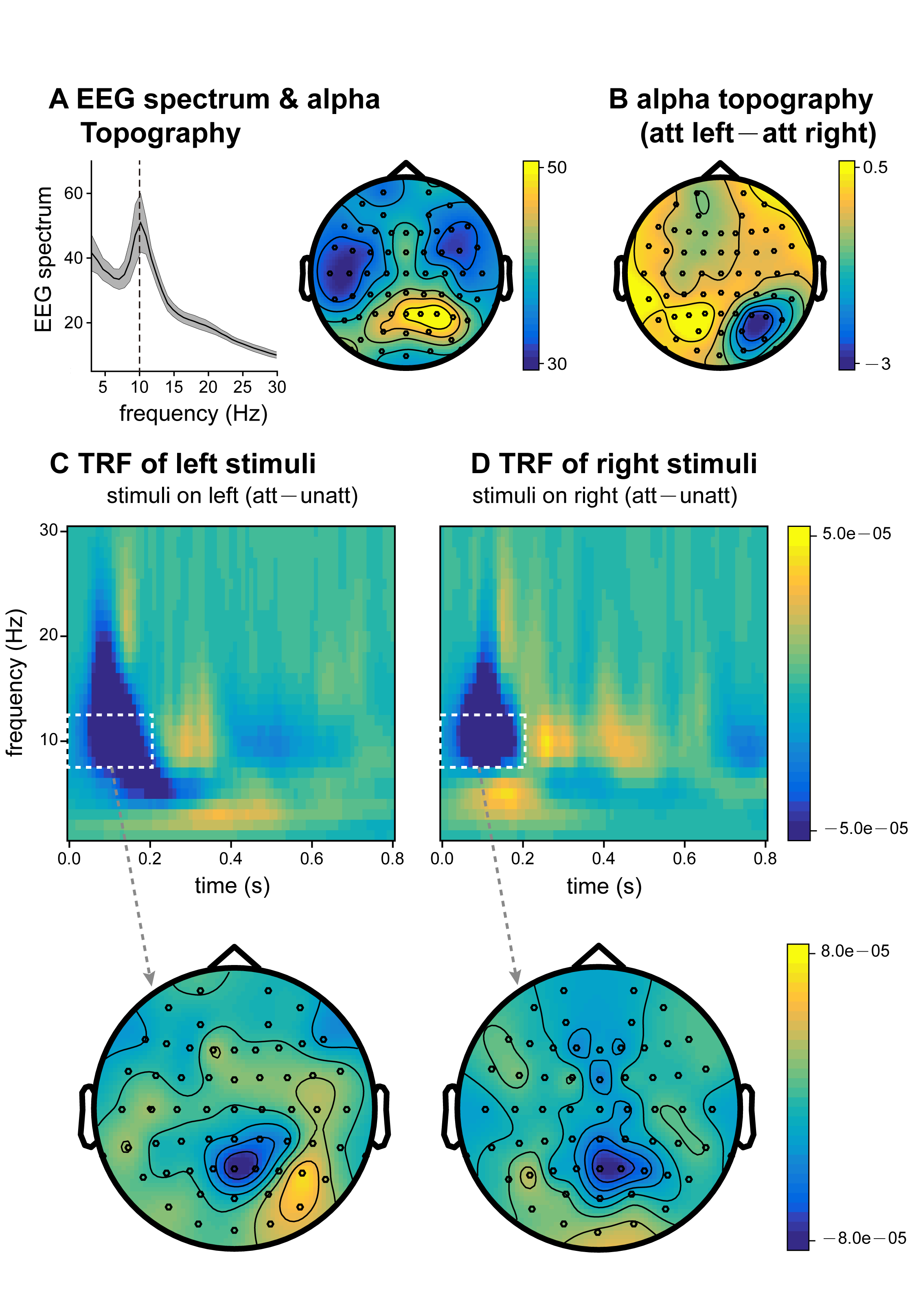

Supplement: S2 Fig — (A) Left: Power spectrum of the EEG during the 5-s stimulus presentation. Right: Spatial distribution of induced alpha-band power. (B) Spatial distribution of induced alpha-band power difference between trials where subjects attended to the left disc and trials where subject attended to the right disc. Note the alpha-band inhibition in contralateral channels, consistent with previous findings. (C) Grand average time-frequency plots for attended (att)–unattended (unatt) TRF power difference (top) and the distribution map for the initial 200 ms alpha-band (dotted white box) inhibition (bottom), for discs presented in the left visual field. (D) Same as C but for discs presented in the right visual field. Note the similar initial alpha-band inhibition and similar spatial distribution to those in Fig 2C & 2D. The data are provided in the Supporting Information (see S6 Data). (TIF) [file pbio.2001903.s002.tif]

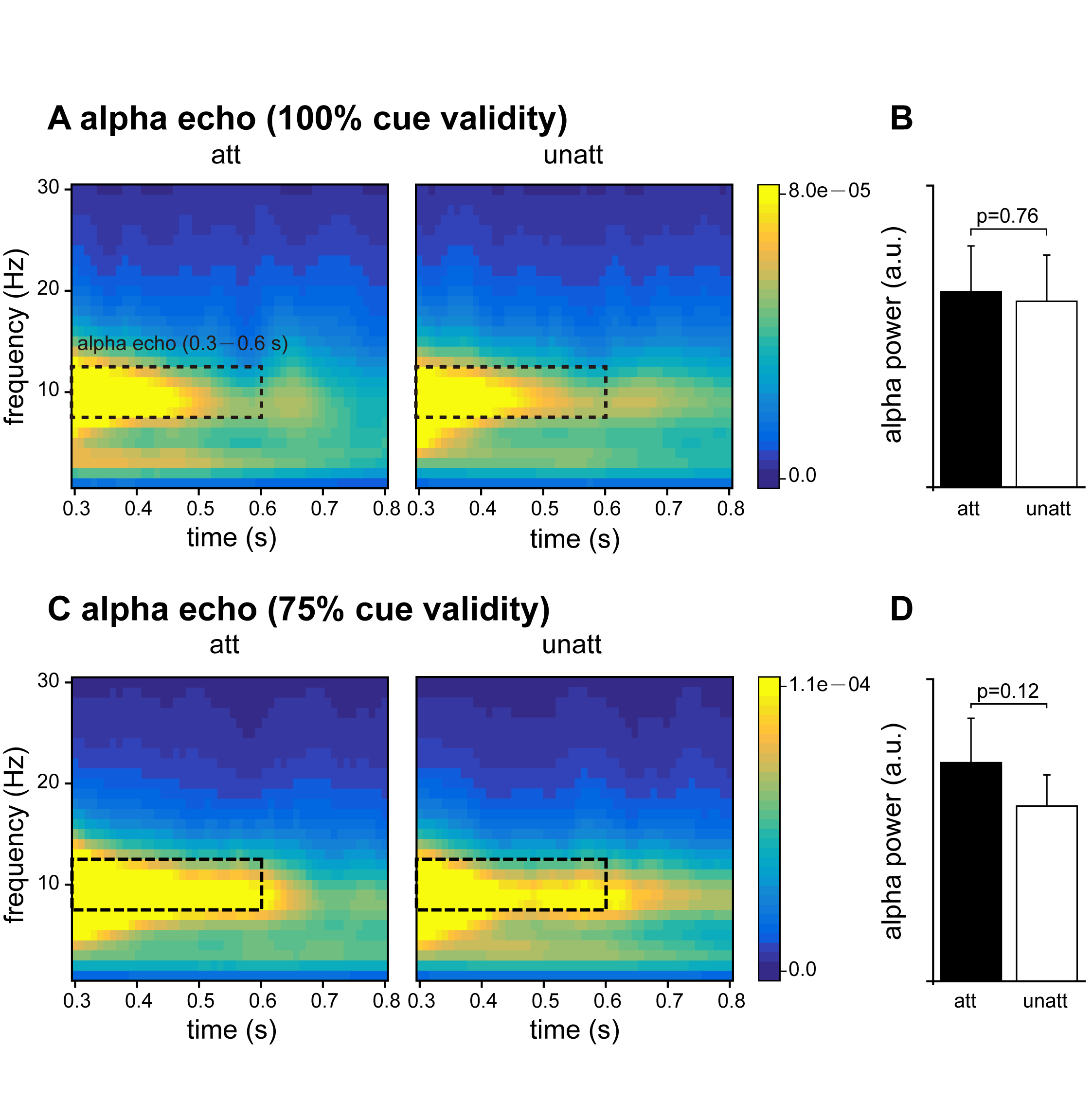

Supplement: S3 Fig — (A) Alpha echoes in Experiment 1. Grand average time-frequency power profile for attended (att) TRF (left) and unattended (unatt) TRF (right), as a function of latency (0.3–0.8 s) and frequency (0–30 Hz). (B) Alpha-echo power (averaged from 0.3–0.6 s, dotted black rectangle) for att (black bar) and unatt (white bar) TRFs. (C) Same as (A) but for Experiment 2. (D) Same as (B) but for Experiment 2. Note the larger overall alpha echoes in both experiments, similar to the results of VanRullen and MacDonald (2012). The data are provided in the Supporting Information (see S7 Data). (TIF) [file pbio.2001903.s003.tif]

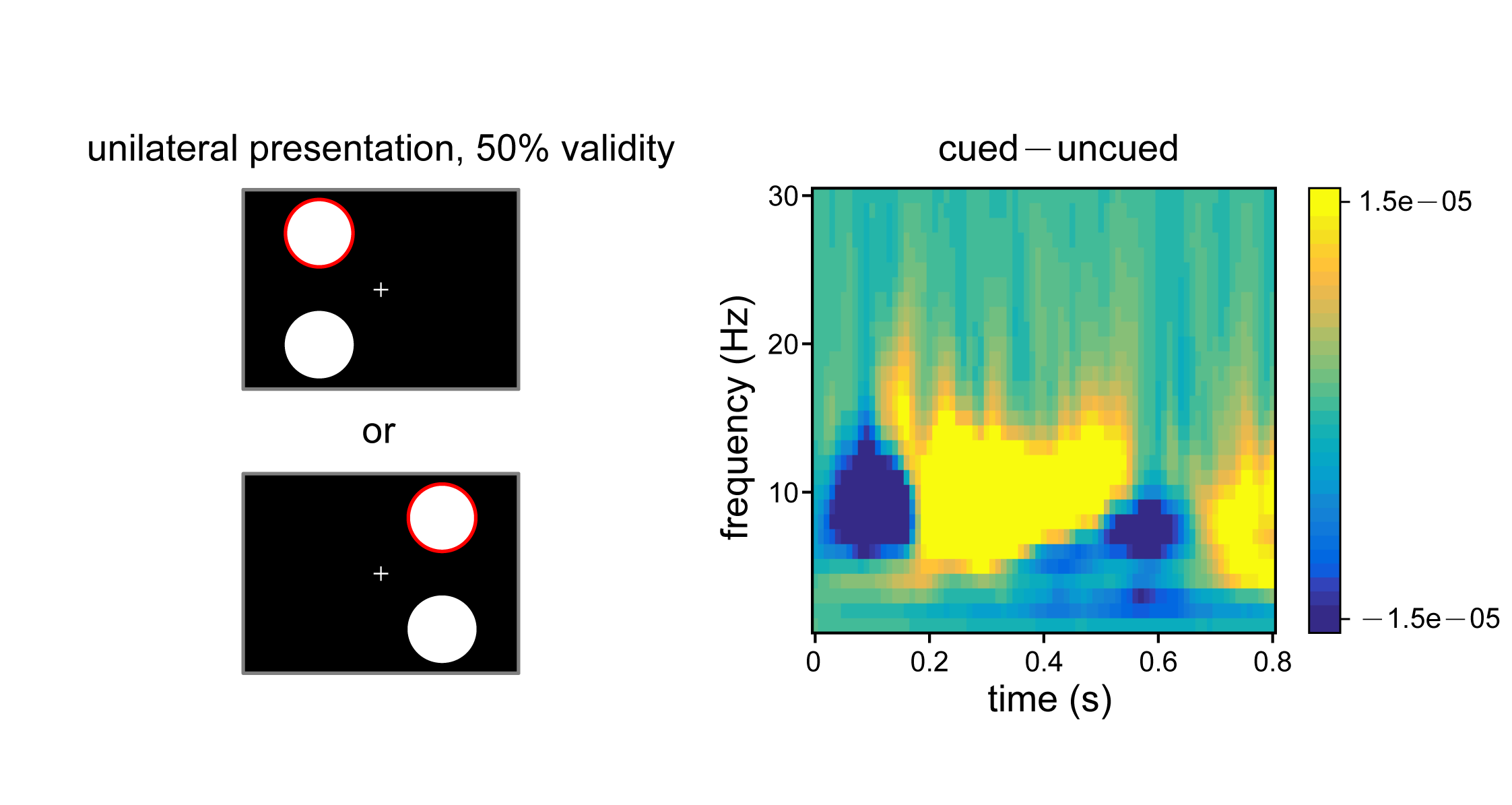

Supplement: S4 Fig — Left: Subjects fixated on a central point and covertly attended to two discs presented in the upper and lower visual field, within the same visual hemifield. Subjects were instructed to pay attention to the two discs simultaneously and were informed that the target was equally likely to appear within both and that the initial cue would not predict the target location. After an uninformative red circle cue (cue validity: 50%) appeared in one of the two discs, the luminance of the two discs was independently and randomly modulated for 5 seconds. Right: Grand average (N = 13) time-frequency plots for cued–uncued TRF power difference. Note the same alpha-band alternating pattern, thus arguing against the interpretation of interhemispheric competition for Experiment 3. The data are provided in the Supporting Information (see S8 Data). (TIF) [file pbio.2001903.s004.tif]

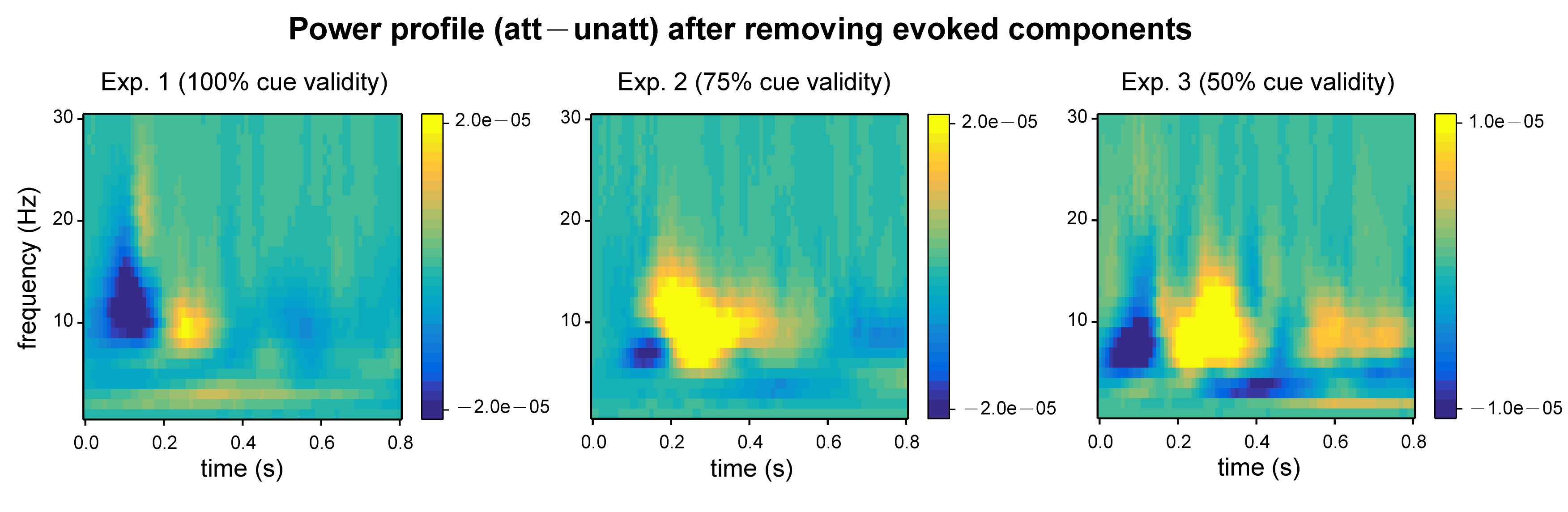

Supplement: S5 Fig — Left: Experiment 1 (cue validity: 100%; N = 18); Middle: Experiment 2 (cue validity: 75%; N = 20); Right: Experiment 1 (cue validity: 50%; N = 16). The data are provided in the Supporting Information (see S9 Data). (TIF) [file pbio.2001903.s005.tif]
